# Influence of Heating Temperature and pH on Acid Gelation of Micellar Calcium Phosphate-Adjusted Skim Milk

**DOI:** 10.3390/foods13111724

**Published:** 2024-05-31

**Authors:** Elaheh Ahmadi, Todor Vasiljevic, Thom Huppertz

**Affiliations:** 1 Advanced Food Systems Research Unit, Institute for Sustainable Industries and Liveable Cities, College of Health and Biomedicine, Victoria University, Melbourne 8001, Australiatodor.vasiljevic@vu.edu.au (T.V.); 2 FrieslandCampina, 3818LE Amersfoort, The Netherlands; 3 Food Quality & Design Group, Wageningen University and Research, 6708WH Wageningen, The Netherlands

**Keywords:** rheology, acid gelation, micellar calcium phosphate, pH adjustment, skim milk

## Abstract

Micellar calcium phosphate (MCP) plays an important role in maintaining the structure and stability of the casein micelle and its properties during processing. The objective of this study was to investigate how heating (10 min at 80 or 90 °C) at different pH levels (6.3, 6.6, 6.9, or 7.2) impacted the acid-induced gelation of MCP-adjusted milk, containing 67 (MCP_67_), 100 (MCP_100_), or 113 (MCP_113_) % of the original MCP content. The unheated sample MCP_100_ at pH 6.6 was considered the control. pH acidification to pH 4.5 at 30 °C was achieved with glucono delta-lactone while monitoring viscoelastic behaviour by small-amplitude oscillatory rheology. The partitioning of calcium and proteins between colloidal and soluble phases was also examined. In MCP-depleted skim milk samples, the concentrations of non-sedimentable caseins and whey proteins were higher compared to the control and MCP-enriched skim milk samples. The influence of MCP adjustment on gelation was dependent on pH. Acid gels from sample MCP_67_ exhibited the highest storage modulus (G′). At other pH levels, MCP_100_ resulted in the greatest G′. The pH of MCP-adjusted skim milk also impacted the gel properties after heating. Overall, this study highlights the substantial impact of MCP content on the acid gelation of milk, with a pronounced dependency of the MCP adjustment effect on pH variations.

## 1. Introduction

Milk is a highly nutritious food containing various proteins, such as caseins and whey proteins, and micronutrients, such as calcium. The stability, nutritional properties, and technological and processing characteristics of milk are mainly influenced by the state of casein micelles and how caseins interact among themselves and with other components [1]. Micellar calcium phosphate (MCP) plays a pivotal role in the structure of casein micelles, acting as a cross-linking bridge and neutralising negatively charged phosphoseryl groups. MCP stands out as a major factor responsible for maintaining the integrity of the micelle [2,3]. Milk processing involves various techniques to stabilise or transform raw milk into a wide range of products [4]. Acid-induced gelation of milk is applied in the production of yogurt and various other fermented dairy products [5], but also takes place during the gastric digestion of milk [6]. The stability of casein micelles in the solution as the building blocks of acid milk gels is governed by steric stabilisation, provided by κ-casein protruding from the surface of the micelles. A reduction in pH leads to a loss of steric stabilisation and the solubilisation of MCP can lead to the coagulation of casein micelles [7].

In the production of fermented products like yogurt, milk is first subjected to heat treatment (e.g., at 80–90 °C for 5–30 min). The purpose of such a high heat treatment is to enhance the textural properties of acid gels, through the denaturation of whey proteins [8]. This denaturation facilitates interactions between whey proteins and caseins, and the heat treatment also causes dissociation of some of the caseins, particularly κ-casein, from casein micelles. These processes contribute to an increased firmness and viscosity of acid-induced gels from heated milk [9]. The impact of composition and processing parameters on the textural properties of acid milk gels has been widely studied in previous studies [9,10,11,12,13,14]. Heat treatment temperature and pH have a significant impact on the texture and physical properties of gels [15]. The intensity of heat treatment influences the extent of whey protein denaturation, thereby affecting the firmness and viscosity of milk [16]. Furthermore, the pH of milk during heat treatment affects the balance between hydrophobic attractions and electrostatic repulsion. Lowering the pH, for instance, shifts the balance toward hydrophobic interactions [3] and it can be possible to produce milk with different levels of whey proteins associated with casein micelles [17]. 

MCP content can influence the functional properties of acid-induced milk gels [18,19]. Limited MCP removal has been noted to support casein micelle structure, potentially improving the formation of crosslinks between strands within the gel network [20]. Over the years, studies have adjusted the content of MCP in both skim milk retentates [21,22] and milk [6,23,24,25,26,27,28,29,30] and examined the impact of MCP adjustment on several important functional properties of milk. It has been found that the levels of individual caseins increase in the serum phase of milk almost linearly with an MCP reduction [31]. In addition, adjusting the MCP content to 67% of its original value results in a high level of intact casein micelles and the greatest thermal stability [30]. 

Previous studies have indicated that modifications in MCP levels within the casein micelle could affect the acid gelation of milk [7,20,32,33,34]. For example, modifying MCP levels by adding different levels of trisodium citrate (TSC) impacted gel stiffness and syneresis in yoghurt [20]. Improved stiffness and reduced syneresis were associated with a low depletion of MCP, which enhanced the rearrangement and molecular mobility of the micelle structure, potentially contributing to increased crosslinking among the strands in yogurt gel networks. Famelart et al. [32] found that a 30% calcium depletion increased the acid gelation pH of milk, while a more extensive depletion led to a decrease in gelation pH. Moreover, Anema [33] reported that partially removing MCP from milk before heat treatment and acidification had minimal impact on gelation pH but significantly reduced the storage modulus, G′. Ozcan et al. [34], on the other hand, elevated the MCP content of milk and noted that it resulted in a limited impact on G′. 

All these findings suggest that the properties of acid-set milk gels are influenced by the level of MCP in the milk undergoing acidification. This study undertook a distinctly different approach, incorporating not only MCP adjustment but also the subsequent adjustment of pH before heating to various levels below and above the natural milk pH, along with exposure to different heating temperatures. The aim of this study was to investigate the influence of heat treatment and pH levels on the acid gelation properties, as well as the protein and mineral distributions, of milk with MCP content adjusted to three levels (67%, 100%, and 113%).

## 2. Materials and Methods

### 2.1. Sample Preparation

Freshly pasteurised skim milk was obtained from Warrnambool Cheese and Butter—Saputo (Warrnambool, Australia). To prevent bacterial growth, 0.02% (*w*/*w*) sodium azide was added. The MCP concentration was adjusted using glucono delta-lactone (GDL) or 1.0 M NaOH to lower or increase the pH of skim milk to 6.1 or 7.5, respectively, thereby adjusting MCP content to 67% (MCP_67_) and 113% (MCP_113_) of the initial MCP content. After stabilising the pH, the dialysis process was carried out as described previously [31]. After MCP content adjustment, the pH of the MCP-adjusted milk was adjusted to 6.3, 6.6, 6.9, or 7.2 through the addition of HCl or NaOH. pH-adjusted samples were heated in an oil bath set at 80 or 90 °C. The temperatures selected are reflective of those commonly used in the pre-heating of milk for yogurt production [9]. The time required to reach these temperatures was approximately 2.5 or 3 min, respectively. Subsequently, the samples were held for a further 10 min at the required temperature, before being cooled to 20 °C by immersion in an ice bath.

**Figure 1:**
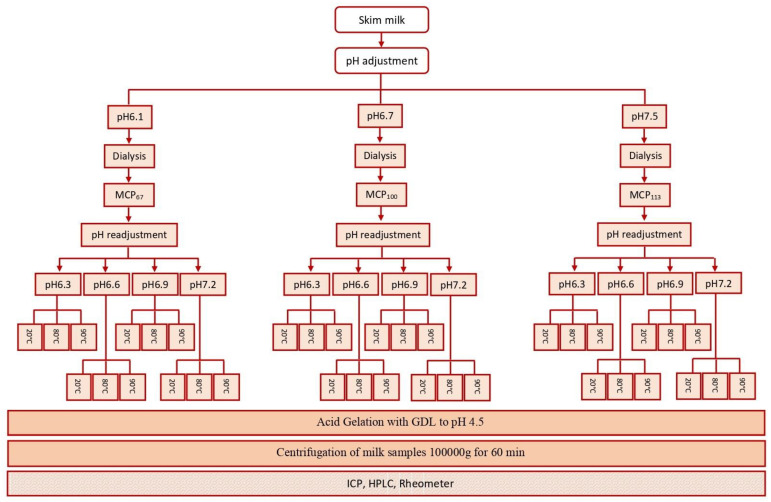
Experimental design of this study.

Acid gelation of unheated and heated pH-adjusted MCP-adjusted skim milk samples was conducted by adding glucono-delta-lactone (GDL) and then incubating the mixture at 30 °C. As the buffering capacity of the milk was altered by changing the MCP levels and pH, the GDL level was varied so that a pH of 4.5 was achieved within a selected time frame; therefore, this GDL amount was established pre-experiments and a pre-determined amount of GDL was added [33]. Incubation was stopped when pH reached 4.5. The experimental design of this study is depicted in Figure 1.

### 2.2. Sample Fractionation

To separate the sedimentable and non-sedimentable phases of skim milk samples before and after heat treatment, ultracentrifugation was performed at 100,000× *g* for 1 h at 20 °C using a Beckman Ultra L-70 centrifuge (Beckman Coulter, Australia Pty., Ltd., Gladesville, Australia). After ultracentrifugation, the clear supernatant from each tube was carefully collected using a syringe [35].

### 2.3. Sample Analysis

#### 2.3.1. Calcium Content

The calcium (Ca) concentrations in the whole samples and serum phases were examined using Inductively Coupled Plasma Atomic Emission Spectrometry (ICP-AES) with an ICPE-9000 system provided by Shimadzu Corporation in Kyoto, Japan, as outlined in a previous paper [30].

#### 2.3.2. High-Performance Liquid Chromatography

Protein levels in milk samples and ultracentrifugal supernatants were examined using reversed-phase high-performance liquid chromatography (RP-HPLC). The RP-HPLC analysis was performed using a Shimadzu HPLC system (Model Prominence-i, LC-2030 C, Shimadzu Corporation, Kyoto, Japan) with a Varian 9012 system controller (Agilent Technologies Inc., Santa Clara, CA, USA). The system was equipped with a refractive index (RI) detector (Varian, 9050) and utilised a C_4_ column (Aeris Widepore, 150 mm × 4.6 mm, 3.6 μm particle size, 300 Å pore size, Phenomenex, Torrance, CA, USA). The analysis was carried out at room temperature [30].

#### 2.3.3. Rheological Measurement

Acid gelation was performed using a controlled-stress rheometer (Physica MCR 301, Anton Paar GmbH, Ostfildern-Scharnhausen, Germany) with a cup (27.11 mm diameter) and bob (25 mm diameter) configuration (CC 25/PR-SN, Anton Paar). The samples were mixed with the required amount of GDL and immediately transferred into the cup. All measurements were performed at a temperature of 30 °C. During the evaluation, the storage modulus (G′) was measured at a strain of 0.5% and a frequency of 1 Hz [36]. The gelation point was identified as the point at which G′ reached a value of 1 Pa [17,37]. Simultaneously, the pH was measured throughout the entire gelation process using a calibrated pH meter equipped with a combined pH electrode featuring a temperature sensor and fixed cable (Model H1131, Hanna Instruments, Woonsocket, RI, USA). These pH measurements were concurrently recorded alongside the rheological measurements [37] to enable the evaluation of G’ as a function of pH.

### 2.4. Statistical Analysis

A randomised, split-plot blocked design was employed for statistical analysis, treated as a General Linear Model with the MCP level serving as the main plot. Subplots included pH adjustment and temperature as factors. The replications were considered as blocks within the design. The dataset underwent analysis using SAS statistical software (version 9.1, SAS Institute, Cary, NC, USA). The predetermined level of significance was set at *p* < 0.05. To enhance reliability, the experimental setup was replicated three times, ensuring robustness and consistency in the obtained results.

## 3. Results

### 3.1. Calcium Distribution

The effect of MCP adjustment, pH, and heat treatment on total calcium and serum calcium is shown in Table 1. Sample MCP_100_ at pH 6.6 and 20 °C had a calcium content of 31.9 mmol L^−1^ and a serum calcium content 10.0 mmol L^−1^ (Table 1). Adjustment of MCP content had a clear influence on the total calcium, as anticipated, with the lowest total calcium level in sample MCP_67_ and the highest in sample MCP_113_ (Table 1). Adjusting pH to 6.3, 6.6, 6.9, or 7.2 did not affect total calcium content before heating all milk samples, while an inverse relation was observed between pH and the amount of soluble calcium (Table 1).

Heating the milk samples at 80 or 90 °C did not impact total calcium but had a substantial impact on the levels of soluble calcium (Table 1). Soluble calcium concentration decreased significantly (*p* < 0.05), by approximately 20–25%, on heating the samples with pH 6.3–6.9 at 80 °C and further decreased (*p* < 0.05) on heating at 90 °C. At pH 6.9, sample MCP_113_ behaved differently compared to other MCP contents, initially following a similar trend—experiencing a decline in soluble calcium after heating at 80 °C but subsequently showing an increase after heating at 90 °C (Table 1). On the contrary, samples adjusted to pH 7.2 exhibited a significant increase (*p* < 0.05) in soluble calcium concentration after heating at 80 °C. However, after heating at 90 °C, the concentration of soluble Ca was either similar to or lower than the initial values. The substantial decrease in the soluble calcium concentration indicated a likely involvement of calcium in complexation with sedimentable proteins.

### 3.2. The Protein Distribution of MCP-Adjusted Skim Milk

The proportions of non-sedimentable caseins and whey proteins in the supernatant of MCP-adjusted skim milk samples relative to the corresponding milk are shown in Table 2 and Table 3, respectively. In sample MCP_100_ at pH 6.6, the proportion of non-sedimentable αs_1_-, αs_2_-, β-, and κ-caseins; α-lactalbumin; and β-lactoglobulin were 3, 13, 18, 13, 95, and 98%, respectively. In unheated samples, a reduction in MCP content resulted in a noticeable increase in the concentrations of non-sedimentable α_s1_-, α_s2_-, β-, and κ-casein (Table 2), while non-sedimentable α-lactalbumin and β-lactoglobulin were not affected (Table 3). In contrast, MCP enrichment significantly (*p* < 0.05) decreased the non-sedimentable levels of αs_2_- and β-caseins, with no observed change in αs_1_- and κ-caseins (Table 2). The pH adjustment to lower pH (6.3, 6.6) slightly elevated the levels of non-sedimentable α_s1_-, α_s2_-, and κ-caseins before heating (Table 2), whereas for non-sedimentable α-lactalbumin and β-lactoglobulin, no change was observed (Table 3).

Heating the samples led to decreased levels of non-sedimentable α_s1_-, α_s2_-, and β-casein (Table 2), as well as non-sedimentable α-lactalbumin and β-lactoglobulin (Table 3), while the level of non-sedimentable κ-casein significantly (*p* < 0.05) increased (Table 2). The changes in the levels of non-sedimentable α_s1_-, α_s2_-, and β-caseins were dependent on both pH and the temperature of heating.

Non-sedimentable αs_1_-casein decreased after heating at 80 and 90 °C, while α_s2_- and β-caseins showed a pH-dependent trend—inverse relationship—with substantial decreases after heating at 80 and 90 °C. In contrast, κ-casein exhibited opposite behaviour from other caseins, with its concentration increasing directly with pH and temperature. The level of non-sedimentable κ-casein in MCP_100_ at elevated pH (6.9 or 7.2) was greater than at lower pH; however, it was lower (*p* < 0.05) than in samples MCP_67_ and MCP_113_ (Table 2).

As expected, heating significantly impacted the whey proteins, particularly at pH 6.9 and 7.2 (Table 3). At pH 6.6 and 6.3, α-lactalbumin appeared more affected than β-lactoglobulin, irrespective of the MCP content (Table 3). A higher temperature (90 °C versus 80 °C) generally led to greater aggregation and lower non-sedimentable α-lactalbumin, except at pH 6.9, where it increased. Non-sedimentable β-lactoglobulin remained consistent across factors, with more retained at lower MCP contents and high pH levels (Table 3). The temperature effect on non-sedimentable β-lactoglobulin might have been confounded by the pH, as it declined at pH 6.3 and 6.6 but increased at pH 6.9 and 7.2 after heating at 90 °C compared to 80 °C (Table 3).

### 3.3. Influence of pH and Heating on Acid Gelation Behaviour of MCP-Adjusted Milk Samples

**Figure 2:**
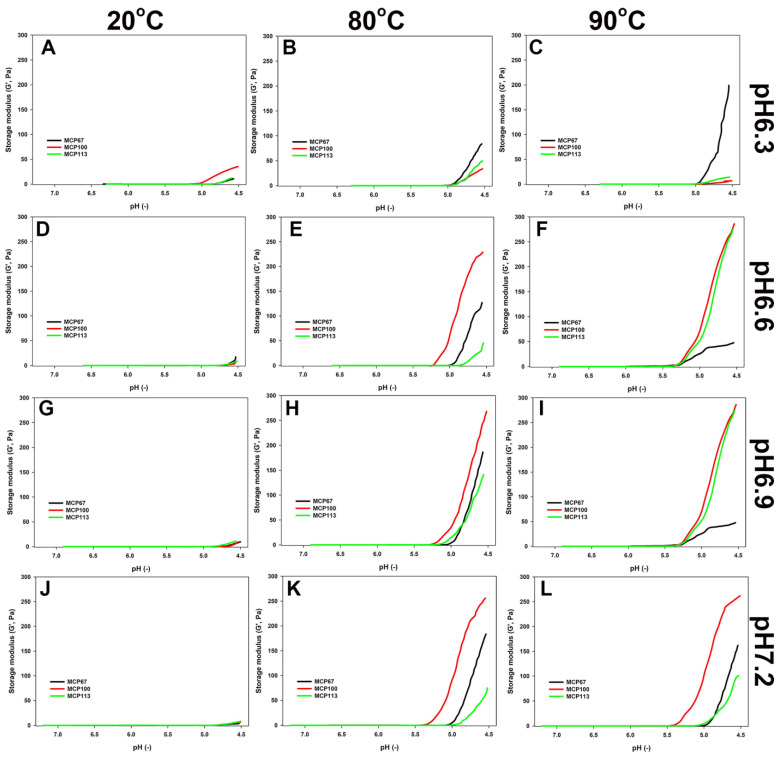
Elastic modulus (G′) as a function of pH during acidification of the skim milk samples with the MCP content adjusted to either 67% (MCP_67_) or 113% (MCP_113_) of its original level (MCP_100_) and pH readjusted to 6.3 (**A**–**C**), 6.6 (**D**–**F**)., 6.9 (**G**–**I**), or 7.2 (**J**–**L**) before (**A**,**D**,**G**,**J**) or after heating 80 (**B**,**E**,**H**,**K**) or 90 (**C**,**F**,**I**,**L**) °C for 10 min. Acidification was achieved by the addition of glucono delta-lactone followed by incubation at 30 °C until pH reached 4.5.

The acid gelation properties of MCP-adjusted skim milk are shown in Table 4. Additionally, Figure 2 shows the evolution of G′ as a function of pH during the GDL-induced acidification of MCP-adjusted milk samples. For unheated milk samples, the MCP content did not affect gelation pH, whereas heat treatment significantly increased gelation pH for most samples (Table 4). The effect of heating temperature showed dependence on the initial pH of the adjusted skim milk in relation to the pH at the gelation point (Table 4). The effects were direct as the samples adjusted to higher pH and heated at the higher temperature started to gel at a higher pH. For example, the samples heated at 90 °C at pH 6.9 or 7.2 began to gel at pH 5.3–5.4, whereas those heated at pH 6.3 started to gel just below pH 5.0 (Table 4).

As shown in Figure 2, the unheated samples gelled less strongly in comparison to the heated samples. The evolution of G′ during acidification was affected by the MCP content, the adjusted pH, and the extent of heat treatment. The acid-induced gels produced by MCP_100_ appeared to have the highest G′. The gel formation of samples MCP_100_ and MCP_113_ was greatly affected at pH 6.3 compared to other pH levels before and after heating. Interestingly, when MCP_100_ was heated at 90 °C with low pH, it formed a gel with a G′ even lower than the sample acidified after heating at 80 °C. After heating at 80 °C, the MCP_67_ samples produced a gel with an appreciable high G′, which was lower than that of the control but greater than that of MCP_113_. The reverse was observed when samples had a pH of 6.6 or 6.9, with MCP_67_ showing impaired gelling behaviour compared to other MCP levels and even to the same MCP level heated at 80 °C (Figure 2E,F,H,I). Gel-forming ability improved somewhat when the pH was adjusted to 6.3 or 7.2 (Figure 2C,L).

## 4. Discussion

Acid-induced gelation of milk is closely linked to pH levels and mineral composition, in particular calcium content and fat and protein content [19,38]. To explore the role of MCP adjustment on acid-induced gelation, three distinct MCP-adjusted skim milk samples—MCP_67_ (33% MCP-depleted), MCP_113_ (13% MCP-enriched), and the control, MCP_100_—were subjected to pH adjustment and heating followed by acidification with GDL at 30 °C in this study. Adjusting the MCP content in skim milk altered the mineral equilibria, significantly impacting the protein distribution between the phases and the properties of caseins in these MCP-adjusted skim milks. Previous studies have investigated the impact of MCP adjustment on milk gelation properties [33,34]. In these studies, only the impact of MCP depletion or the enrichment of skim milk on acid-induced gelation was examined. In the present work, however, a different approach was employed; in addition to MCP adjustment, it involved the adjustment of pH to various levels below and above that of the natural milk pH and subjecting such milk to different heating temperatures.

The adjustment of MCP content changes the calcium equilibrium in milk (Table 1), resulting in a significant alteration in the amount of non-sedimentable caseins (Table 2), which likely depicts a partial dissociation of individual caseins from the micelle, which confirmed previous findings [6,25,29,30,31]. Variations in MCP content also influenced the properties of the casein micelles. The stability of the casein micelle primarily relies on the steric stabilisation by a layer of κ-casein, often described as a salted polyelectrolyte brush [39]. The κ-casein level in the serum phase of the milk sample (MCP_100_) from the control group was determined to be minimal. However, as the MCP content decreased, the level of κ-casein in the serum phase increased (Table 2). Reducing MCP content increases the concentration of non-micellar caseins in the serum phase, whereas an increase in the MCP content induces a transition of individual caseins from the soluble phase into the micelles (Table 2) [31]. A consistent proportion of non-sedimentable κ-casein in the MCP-enhanced skim milk across the whole pH range compared to MCP_100_ (Table 2) suggests that its concentration may not be entirely dependent on the MCP content. pH adjustment before heating has also affected the distribution of soluble calcium and caseins. Lowering the pH to 6.3 and 6.6 increased the dissociation of calcium and non-sedimentable caseins from the casein micelle into the serum phase, which is illustrated in Table 1 and Table 2, and supported by our previous findings [29,30].

The initial pH of skim milk during heating has a crucial impact on the acid-induced gel. Anema et al. [17] and Lucey et al. [15] demonstrated that, at pH levels equal to or greater than 7.0, only minimal amounts of denatured whey proteins are associated with the casein micelles, which is supported by a higher level of κ-casein, α-lactalbumin, and β-lactoglobulin in the serum phase (Table 2 and Table 4) and a slightly lower G′ of acid gels from all samples made from milk samples adjusted to pH 7.2 (Figure 2) in our study. Heating at pH 6.3 led to a lower value of G′ in these acid gels (Figure 2), indicating weaker intramolecular interactions. As the pH decreases, MCP progressively solubilises, causing the removal of residual MCP linkages from the gel structure during its formation [33]. Interestingly, the content of MCP had only a slight effect on the dissociation of k-casein following heating (Table 2). In addition, the distribution of whey proteins between the sedimentable and non-sedimentable phases closely followed that of k-casein (Table 3).

Moreover, the partial removal of MCP from the milk (MCP-depleted skim milk) before heat treatment and acidification significantly decreased the G′. The G′ modulus of gels correlates with the quantity, strength, or number of bonds among casein particles, as well as the arrangement of casein strands in the network [40]. In addition, dissolving MCP within casein particles leads to a decrease in MCP crosslinks and potentially heightens electrostatic repulsion among exposed phosphoserine residues [16]. Both of these factors could significantly contribute to the decrease in G′ values observed in MCP-depleted acidified milk. Ozcan-Yilsay et al. [20] reported findings that agree with the current observations, outlining that the removal of MCP from the milk before heat treatment and acidification significantly decreased the G′. In contrast, Anema [33] reported that reducing the MCP level of milk increased the G’ of acid gels therefrom. While these outcomes might be conflicting, they could also be attributed to the differing methods used to alter MCP levels.

Furthermore, surprisingly, the effects of MCP adjustment demonstrated a notable dependence on pH in cases where heat treatment was applied. The acidification of MCP_67_ at pH 6.3 exhibited a pronounced rise in G′, particularly after heating at 90 °C compared to other MCP-adjusted samples (Figure 2B,C). The possible reason for this observation is that the solubilisation of MCP during MCP adjustment results in a reduction in MCP crosslinks, prompting the dissociation of caseins from casein micelles (Table 2) [2,20]. The key distinction lies in the increased presence of non-sedimentable caseins, which are a consequence of reduced MCP content and may influence the aggregation behaviour of whey proteins. For instance, when samples with lower MCP content were heated at pH 6.3, there was less incorporation of β-lactoglobulin and α-lactalbumin into sedimentable aggregates (Table 3). As whey proteins require interaction with κ-casein to form bridges with the casein micelle, this leads to a greater G’ in a lower MCP content. However, the acidification of heated MCP-depleted skim milk at higher pH levels (6.6, 6.9, 7.2) indicated minimal gel formation in MCP-depleted skim milk samples.

Nevertheless, at most other pH levels, samples containing 100% MCP demonstrated the highest G′ increase and firmer gels. This observation highlights the nuanced influence of MCP concentration on pH variations, emphasising the critical interplay between these factors in determining the gelation properties. Conflicting views exist regarding the impact of MCP enhancement on acid gelation properties. Anema [33] reported that higher MCP levels contribute to the formation of a more elastic gel, whereas Ozcan et al. [34] proposed that an increase in MCP content had a limited impact on G′. The present study suggested that the effect of MCP enhancement depends on the initial pH of skim milk and the heat treatment temperature; after heat treatment at 90° for MCP-enhanced skim milk at pH 6.6 and 6.9, G′ showed the highest increase.

## 5. Conclusions

Adjusting MCP levels alters the total calcium content in skim milk, while pH adjustment impacts the soluble calcium. Both non-sedimentable caseins and whey proteins are influenced by changes in MCP levels and pH. However, the behaviour of non-sedimentable κ-caseins may not be solely dependent on MCP content and might be affected by other electrostatic interactions. Additionally, heat treatment significantly influences the protein and calcium balance, leading to different properties in acid-induced gels. Higher temperatures cause calcium phosphate solubilisation, whey protein denaturation, and increased gel firmness. The initial pH of skim milk during heating is crucial as it affects the solubilisation of micellar calcium phosphate and, consequently, gel strength. This study highlights the nuanced effects of MCP concentration and pH variations on gelation properties. Overall, this comprehensive investigation sheds light on the intricate interplay of factors affecting acid-induced gelation, contributing valuable insights for the dairy industry.

## Figures and Tables

**Table 1:** Calcium concentration of the bulk and serum phase of pasteurised skim milk with their MCP adjusted to 67% (MCP_67_) or 113% (MCP_113_) of their original level (MCP_100_) by either acidification or alkalisation followed by exhaustive dialysis against bulk milk followed by pH readjustment to 6.3, 6.6, 6.9, or 7.2 before and after heat treatment at 80 or 90 °C for 10 min. For sample details, see Figure 2.

Sample	pH	Calcium Concentration (mmol L^−1^)					
		Milk			Serum		
		20 °C	80 °C	90 °C	20 °C	80 °C	90 °C
MCP_67_	6.3	24.8 ^Ad^	24.2 ^Ad^	24.7 ^Ad^	11.5 ^Ab^	9.1 ^Bb^	8.8 ^Ba^
	6.6	25.5 ^Ad^	25.2 ^Ad^	24.8 ^Ad^	10.0 ^Ac^	7.8 ^Bd^	6.9 ^Cd^
	6.9	26.7 ^Ac^	26.2 ^Ac^	26.2 ^Ac^	8.8 ^Ae^	6.7 ^Bg^	5.1 ^Ci^
	7.2	25.7 ^Ac^	26.0 ^Ac^	25.6 ^Ac^	5.5 ^Bi^	6.8 ^Ag^	5.4 ^Bh^
MCP_100_	6.3	31.9 ^Ab^	31.8 ^Ab^	31.6 ^Ab^	13.4 ^Aa^	10.6 ^Ba^	7.9 ^Cc^
	6.6	31.9 ^Ab^	31.9 ^Ab^	31.2 ^Ab^	10.0 ^Ac^	8.3 ^Bc^	8.7 ^Ba^
	6.9	30.9 ^Ab^	31.2 ^Ab^	31.6 ^Ab^	8.4 ^Af^	7.2 ^Be^	6.9 ^Bd^
	7.2	31.4 ^Ab^	31.7 ^Ab^	31.0 ^Ab^	7.2 ^Ah^	7.8 ^Ad^	6.2 ^Bf^
MCP_113_	6.3	34.2 ^Aa^	34.1 ^Aa^	34.4 ^Aa^	9.6 ^Ad^	8.2 ^Bc^	8.3 ^Bb^
	6.6	35.1 ^Aa^	34.5 ^Aa^	34.9 ^Aa^	8.0 ^Ag^	7.0 ^Bf^	6.6 ^Ce^
	6.9	35.3 ^Aa^	34.3 ^Aa^	34.5 ^Aa^	5.6 ^Bi^	4.9 ^Bh^	6.3 ^Af^
	7.2	34.6 ^Aa^	34.2 ^Aa^	34.6 ^Aa^	5.3 ^Bj^	7.8 ^Ad^	5.8 ^Bg^

Lowercase superscript letters indicate significant differences in columns (*p* < 0.05). Uppercase superscript letters signify the differences in the rows for the milk and for the serum separately (*p* < 0.05).

**Table 2:** The proportion of non-sedimentable caseins, expressed as a percentage of caseins in the bulk sample, of MPC-adjusted skim milk adjusted to pH 6.3, 6.6., 6.9, or 7.2 before heating at 80 or 90 °C for 10 min.

Sample	pH	Milk Protein Concentration (%)											
		α_s1_-Casein			α_s2_-Casein			β-Casein			κ-Casein		
		20 °C	80 °C	90 °C	20 °C	80 °C	90 °C	20 °C	80 °C	90 °C	20 °C	80 °C	90 °C
MCP_67_	6.3	11.3 ^Aa^	5.6 ^Bc^	5.7 ^Ba^	21.2 ^Ab^	5.7 ^Bd^	3.7 ^Cf^	36.9 ^Aa^	21.3 ^Ba^	18.9 ^Ba^	26.0 ^Aa^	23.1 ^Bd^	26.9 ^Ad^
	6.6	10.6 ^Aa^	6.7 ^Bb^	5.8 ^Ba^	23.4 ^Aa^	5.8 ^Bd^	5.0 ^Be^	36.4 ^Aa^	19.3 ^Bb^	15.2 ^Cb^	15.4 ^Bb^	24.1 ^Ad^	24.9 ^Ae^
	6.9	8.0 ^Ab^	5.0 ^Bc^	5.8 ^Ba^	17.1 ^Ac^	1.3 ^Bf^	1.3 ^Bg^	36.6 ^Aa^	12.3 ^Bd^	10.5 ^Ccd^	13.6 ^Cbc^	29.5 ^Bbc^	40.8 ^Ab^
	7.2	8.8 ^Ab^	7.8 ^Ba^	5.8 ^Ca^	10.1 ^Af^	3.3 ^Be^	2.0 ^Cg^	36.3 ^Aa^	12.9 ^Bd^	11.4 ^Cc^	12.5 ^Ccd^	28.1 ^Bc^	37.0 ^Ac^
MCP_100_	6.3	5.5 ^Ad^	2.1 ^Cf^	3.8 ^Bb^	14.8 ^Ad^	9.3 ^Bb^	8.4 ^Bc^	18.0 ^Ac^	12.8 ^Bd^	10.0 ^Cd^	10.2 ^Be^	20.0 ^Ae^	19.7 ^Af^
	6.6	2.9 ^Be^	2.0 ^Cf^	4.0 ^Ab^	12.9 ^Ae^	7.5 ^Bc^	6.6 ^Bd^	17.9 ^Ac^	13.1 ^Bd^	10.0 ^Cd^	12.6 ^Bc^	17.7 ^Af^	19.4 ^Af^
	6.9	3.1 ^Ae^	2.9 ^Aef^	2.5 ^Ac^	15.6 ^Acd^	7.2 ^Bcd^	1.7 ^Cg^	19.9 ^Ab^	7.7 ^Be^	7.7 ^Be^	10.9 ^Ccd^	30.2 ^Bb^	35.8 ^Ac^
	7.2	2.2 ^Ce^	6.7 ^Ab^	3.7 ^Bb^	8.3 ^Ag^	4.4 ^Bde^	1.0 ^Cgh^	12.1 ^Ae^	10.1 ^Bd^	8.5 ^Cde^	10.2 ^Ce^	28.9 ^Bbc^	35.8 ^Ac^
MCP_113_	6.3	5.7 ^Ad^	4.1 ^Bcd^	3.2 ^Bbc^	11.9 ^Ce^	15.8 ^Aa^	13.6 ^Ba^	13.4 ^Bde^	15.1 ^Ac^	11.4 ^Cc^	11.1 ^Ccd^	18.5 ^Bef^	23.9 ^Ae^
	6.6	6.7 ^Ac^	5.2 ^Bc^	4.0 ^Cb^	7.1 ^Bg^	9.6 ^Ab^	11.0 ^Ab^	14.9 ^Ad^	13.5 ^Bcd^	10.4 ^Ccd^	12.7 ^Cc^	19.8 ^Be^	23.9 ^Ae^
	6.9	2.3 ^Ae^	2.3 ^Af^	1.5 ^Ad^	2.5 ^Ah^	2.2 ^ABef^	0.7 ^Bgh^	10.2 ^Af^	7.0 ^Be^	6.3 ^Bef^	10.5 ^Ce^	33.1 ^Ba^	44.4 ^Aa^
	7.2	3.6 ^Ae^	3.4 ^Ade^	1.4 ^Bd^	9.5 ^Afg^	1.1 ^Bf^	0.0 ^Bh^	17.3 ^Ac^	8.0 ^Be^	7.3 ^Be^	10.5 ^Ce^	34.0 ^Ba^	43.7 ^Aa^

Lowercase superscript letters indicate significant differences in columns (*p* < 0.05). Uppercase superscript letters signify the differences in the rows for each casein separately (*p* < 0.05).

**Table 3:** The proportion of non-sedimentable whey proteins, expressed as a percentage of whey proteins in the bulk sample, in MPC-adjusted skim milk adjusted to pH 6.3, 6.6., 6.9, or 7.2 before heating at 80 or 90 °C for 10 min.

Sample	pH	Whey Protein Concentration (%)					
		α-Lactalbumin			β-Lactoglobulin		
		20 °C	80 °C	90 °C	20 °C	80 °C	90 °C
MCP_67_	6.3	86.8 ^Ac^	21.6 ^Be^	20.3 ^Bf^	91.4 ^Ab^	45.2 ^Bcd^	42.9 ^Bde^
	6.6	87.7 ^Abc^	30.6 ^Bd^	24.2 ^Be^	91.7 ^Ab^	54.2 ^Bb^	39.4 ^Cef^
	6.9	84.5 ^Acd^	76.6 ^Ba^	79.3 ^Ba^	97.8 ^Aa^	60.8 ^Ba^	56.1 ^Bb^
	7.2	81.9 ^Ae^	67.6 ^Bb^	60.4 ^Bd^	98.4 ^Aa^	53.7 ^Bb^	56.4 ^Bb^
MCP_100_	6.3	89.3 ^Ab^	10.7 ^Bg^	10.5 ^Bh^	97.8 ^Aa^	39.9 ^Ce^	61.8 ^Ba^
	6.6	94.6 ^Aa^	23.6 ^Be^	17.1 ^Cg^	98.4 ^Aa^	37.1 ^Cf^	59.4 ^Ba^
	6.9	95.4 ^Aa^	62.5 ^Cc^	67.0 ^Bb^	95.3 ^Aa^	43.9 ^Bd^	44.6 ^Bd^
	7.2	91.3 ^Ab^	60.4 ^Cc^	63.4 ^Bc^	97.9 ^Aa^	32.7 ^Cg^	41.5 ^Be^
MCP_113_	6.3	91.1 ^Ab^	18.1 ^Bf^	10.2 ^Ch^	83.2 ^Ac^	39.0 ^Bef^	37.0 ^Bf^
	6.6	85.6 ^Ac^	36.0 ^Bd^	25.2 ^Ce^	78.8 ^Ad^	34.6 ^Bfg^	22.6 ^Cg^
	6.9	82.3 ^Ade^	68.3 ^Bb^	63.7 ^Cc^	97.1 ^Aa^	41.5 ^Cde^	48.6 ^Bc^
	7.2	85.0 ^Ac^	68.6 ^Bb^	60.6 ^Cd^	79.7 ^Ad^	47.4 ^Bc^	44.7 ^Bd^

Lowercase superscript letters indicate significant differences in columns (*p* < 0.05). Uppercase superscript letters signify the differences in the rows for each whey protein separately (*p* < 0.05).

**Table 4:** Time and pH at gelation point during acid-induced gelation of skim milk with MCP content adjusted to either 67 (MCP_67_) or 113% (MCP_113_) of its original level (MCP_100_) with the pH readjusted to 6.3, 6.6., 6.9, or 7.2 before and after heat treatment at 80 or 90 °C for 10 min.

Sample	pH	pH at Gelation Point (-)		
		Temperature (°C)		
		20	80	90
MCP_67_	6.3	4.81 ^Ab^	4.96 ^Abc^	4.97 ^Abc^
	6.6	4.71 ^Bb^	4.98 ^Abc^	4.92 ^ABbc^
	6.9	4.70 ^Cb^	5.01 ^Bbc^	5.29 ^Aab^
	7.2	4.76 ^Cb^	5.02 ^Bbc^	5.40 ^Aa^
MCP_100_	6.3	5.05 ^Aa^	4.98 ^Abc^	4.79 ^Bc^
	6.6	4.66 ^Bb^	5.13 ^Aab^	5.22 ^Aab^
	6.9	4.77 ^Bb^	5.25 ^Aab^	5.43 ^Aa^
	7.2	4.79 ^Bb^	5.36 ^Aa^	5.44 ^Aa^
MCP_113_	6.3	4.81 ^Ab^	4.95 ^Abc^	4.95 ^Abc^
	6.6	4.70 ^Bb^	5.06 ^Ab^	4.96 ^Abc^
	6.9	4.82 ^Bb^	5.16 ^Aab^	5.33 ^Aab^
	7.2	4.77 ^Bb^	4.96 ^ABbc^	5.09 ^Ab^

Lowercase superscript letters indicate significant differences in columns (*p* < 0.05). Uppercase superscript letters show differences in the rows for time and pH at the gelation point separately (*p* < 0.05).

## Data Availability

The original contributions presented in the study are included in the article, further inquiries can be directed to the corresponding author.
